# Lichen Specific Thallus Mass and Secondary Compounds Change across a Retrogressive Fire-Driven Chronosequence

**DOI:** 10.1371/journal.pone.0049081

**Published:** 2012-11-08

**Authors:** Johan Asplund, Aron Sandling, David A. Wardle

**Affiliations:** 1 Department of Forest Ecology and Management, Swedish University of Agricultural Sciences, Umeå, Sweden; 2 Department of Ecology and Natural Resource Management, Norwegian University of Life Sciences, Ås, Norway; USDA-ARS, United States of America

## Abstract

In the long-term absence of major disturbances ecosystems enter a state of retrogression, which involves declining soil fertility and consequently a reduction in decomposition rates. Recent studies have looked at how plant traits such as specific leaf mass and amounts of secondary compounds respond to declining soil fertility during retrogression, but there are no comparable studies for lichen traits despite increasing recognition of the role that lichens can play in ecosystem processes. We studied a group of 30 forested islands in northern Sweden differing greatly in fire history, and collectively representing a retrogressive chronosequence, spanning 5000 years. We used this system to explore how specific thallus mass (STM) and carbon based secondary compounds (CBSCs) change in three common epiphytic lichen species (*Hypogymnia phsyodes, Melanohalea olivacea* and *Parmelia sulcata*) as soil fertility declines during this retrogression. We found that STMs of lichens increased sharply during retrogression, and for all species soil N to P ratio (which increased during retrogression) was a strong predictor of STM. When expressed per unit area, medullary CBSCs in all species and cortical CBSCs in *P. sulcata* increased during retrogression. Meanwhile, when expressed per unit mass, only cortical CBSCs in *H. physodes* responded to retrogression, and in the opposite direction. Given that lichen functional traits are likely to be important in driving ecological processes that drive nutrient and carbon cycling in the way that plant functional traits are, the changes that they undergo during retrogression could potentially be significant for the functioning of the ecosystem.

## Introduction

As ecosystems develop during succession they undergo an initial build-up phase which is characterized by an accumulation of nutrients, leading to a maximum biomass phase [1]–[3]. However, in the long-term absence of major disturbances over thousands or more years, increasing nutrient limitation frequently leads to a decline in plant productivity and standing biomass, and corresponding impairment of belowground processes [4]–[6]. Chronosequences that include retrogressive stages are amongst some of the most extreme gradients of soil fertility that exist across otherwise comparable ecosystems with similar climate and geology, and are therefore powerful study systems for investigating the effects of large shifts in soil fertility on ecological properties [5]–[7]. As such, a growing number of studies have used retrogressive chronosequence for exploring how plant functional traits change as soil fertility declines over time [8]–[13]. However, the response of functional traits of non-vascular life forms such as lichens, which play a prominent role in many forested ecosystems worldwide, remains unexplored.

Unlike vascular plants, lichens do not physiologically control their water relations. Instead, lichens can improve their water economy by increasing their specific thallus mass (STM, biomass per unit area), especially by thickening the photobiont layer [14]. The STM is an important functional trait that is directly or indirectly controlled by exposure to solar radiation [15], [16], and considerable changes in STM occur within a few months after alteration of light exposure in lichen transplantation experiments [17], [18]. Further, STM could potentially have afterlife effects by controlling lichen litter quality and decomposability, as has been shown for comparable measures for vascular plants such as specific leaf mass (SLM, leaf mass per unit area; the reciprocal of specific leaf area) [19], [20]. Previous studies have shown SLM to increase as soil fertility declines such as occurs during retrogression [11], [21], but whether STM for lichens shows similar responses remains unexplored.

**Figure 1:**
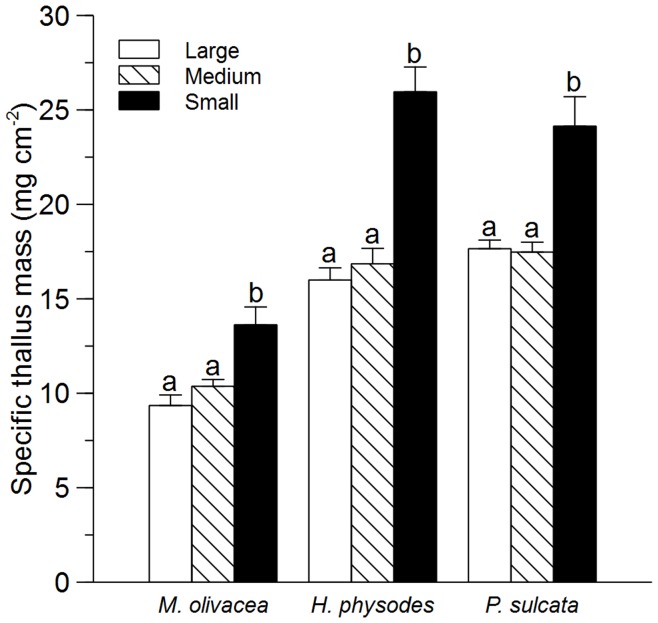
Response of lichen specific thallus mass to island size. Values are given for mean+SE of *M. olivacea*, *H. physodes* and *P. sulcata* on large small, medium and small sized islands. Letters on top of bars denotes significant differences (p<0.001, Tukey’s *post hoc* test) within species. ANOVA results given in table 1.

Many lichens produce carbon based secondary compounds (CBSCs), most of which are weak phenolic acid derivatives. These compounds protect the lichens against biotic and abiotic stresses [22] and usually make up 1–5% (but occasionally as high as 30%) of the lichen biomass [23]. Light-screening secondary compounds in the lichen cortex have been shown to increase when light levels are increased [24], [25]. Meanwhile, secondary compounds in the lichen medulla, which do not play a photoprotective role, seem to be less responsive to solar radiation [26], although both positive [16] and negative [27] responses have been reported. Further, Vatne et al. [28] showed that medullary compounds in the lichen *Lobaria pulmonaria* are also responsive to other extrinsic factors such as altitude and increasing soil pH. However, although secondary compound production in vascular plants is known to increase during declines in soil fertility associated with retrogression [10], [29], the impact of soil infertility on CBSCs has not been investigated.

**Table 1:** ANOVA results for island size and species effects on lichen traits.

	STM		Medullary secondary compounds				Cortical secondary compounds				
			per mass		per area		per mass		per area		
	*F*	*P*	*F*	*P*	*F*	*P*	*F*	*P*	*F*	*P*	
Island Size (IS)	**29.71**	**<0.001**	1.597	0.221	**13.95**	**<0.001**	**5.419**	**0.011**	**7.125**	**0.003**	
Species (S)	**168.5**	**<0.001**	**291.7**	**<0.001**	**282.9**	**<0.001**	**15.15**	**<0.001**	**6.574**	**0.016**	
IS×S	**6.618**	**<0.001**	0.801	0.530	**9.479**	**<0.001**	**9.538**	**<0.001**	1.351	0.276	

Results are from two-way split-plot ANOVAs testing for the effect of island size class (large, middle, small) as the main plot factor, and lichen species (*M. olivacea, H. physodes* and *P. sulcata*) as the sub-plot factor on specific thallus mass (STM) and medullary and cortical secondary compounds measured per unit mass as well as unit area.

df: IS = 2, 27, S = 2, 54, IS×S = 4, 54, except df for cortical compounds: IS = 2, 27, S = 1, 27, IS×S = 2, 27.

*M. olivacea* not included in ANOVAs for cortical compounds because all values were zero.

In this study, we used a gradient of 30 islands comprising a chronosequence generated by increasing time since fire and spanning 5000 years [12]. In this system, larger islands get struck by lightning more often than smaller ones, so as island size declines the mean time since the most recent fire increases [30], [31]. With increasing time since fire the islands enter into a state of ecosystem ‘retrogression’, accompanied by a decline in plant productivity, soil fertility and litter decomposition rates [12], [30]. In the present study we investigated how STM and CBSCs in three common epiphytic lichens vary across this retrogressive island chronosequence. We have chosen to sample lichens on trunks of *Betula pubescens*, a deciduous tree species that shows considerable changes in leaf traits and secondary compound production as retrogression proceeds [10], [11], [13]. On these islands, the trunks of *B. pubescens* host approximately 20 lichen species, and in the present study we have focused on three lichen species that occur on all islands and collectively comprise 70% of the lichen cover on *B. pubescens* trunks, i.e., *Hypogymnia physodes*, *Melanohalea olivacea* and *Parmelia sulcata*. We predict that ecosystem retrogression will cause STM and CBSC concentrations in epiphytic lichens to increase. Because these thallus traits could potentially influence lichen tissue decomposition rates and consumption by invertebrates and thus the turnover rates of carbon and nutrients [32]–[34], our study contributes to the understanding of how long-term ecosystem development may influence the contribution of lichens to community and ecosystem processes [35], [36].

**Table 2:** Relationships between lichen traits and environmental variables.

	*M. olivacea*		*H. physodes*				*P. sulcata*			
	STM	Medullary per area	STM	Corticalper mass	Corticalper area	Medullaryper area	STM	Corticalper mass	Corticalper area	Medullaryper area
STM	**−**	0.752***	**−**	−0.830***	0.556**	0.815***	**−**	−0.489**	0.350	0.652***
Bark pH	**0.401** *	0.359	0.502**	−0.598***	0.125	0.500**	0.625***	−0.010	0.583***	**0.462** *
Bark roughness	−0.218	−0.281	−0.295	**0.428** *	**−**0.069	**−0.389** *	−0.327	0.359	−0.096	−0.475**
DBH	−0.302	−0.180	**−0.443** *	0.599***	0.006	**−0.457** *	**−0.437** *	−0.008	−0.228	−0.327
Humus depth	0.621***	**0.445** *	0.627***	−0.574***	0.359	**0.415** *	**0.393** *	−0.137	0.310	**0.460** *
Light	0.318	0.317	**0.383** *	−0.519**	−0.024	0.343	0.516**	−0.220	0.318	0.323
Productivity	−0.529**	**−0.453** *	−0.686***	0.677***	−0.292	−0.495**	−0.515**	0.052	**−0.453** *	−0.351
Soil N:P	0.626***	**0.417** *	0.632***	−0.579***	0.344	0.490**	0.465**	−0.196	0.302	0.491**
Soil pH	−0.199	0.060	**−0.377** *	0.247	−0.219	−0.215	−0.134	0.219	−0.010	−0.211
Soil total N	**0.436** *	0.256	**0.402** *	**−0.451** *	0.175	0.259	**0.372** *	−0.149	0.191	**0.390** *
Soil total P	−0.479**	−0.324	−0.552**	**0.395** *	**−0.362** *	−0.513**	−0.354	0.214	**−0.362** *	−0.268

Values given are Spearman rank correlations between specific thallus mass (STM) and medullary and cortical secondary compounds expressed as per thallus mass as well as per thallus area of three lichens species and a number of environmental variables measured in the soil and on the host tree (*B. pubescens*) across gradient of 30 islands differing in size. *n* = 30 islands.

* , ** and *** denotes *P* = 0.05, 0.01 and 0.001, respectively.

Medullary compounds per unit mass never showed a correlation with any environmental variable and are therefore not included.

## Materials and Methods

### Ethics Statement

We used 30 forested islands located in the two neighbouring lakes, Hornavan and Uddjaur, in the northern boreal zone of Sweden (65° 55′ N; 17° 43′ E to 66° 09′ N; 17° 55′ E). These islands are not within national reserves, and the land is public and not government protected. We confirm that all national and international rules were complied with during the field work. The research did not involve measurements on humans or animals. The plant material collected for this study was only sampled at a very limited scale and therefore had negligible effects on broader ecosystem functioning. We have no commercial interests or conflicts of interest in performing this work.

**Table 3:** ANOVA results for properties of *B. pubescens* measured across the three island size classes.

	Large	Medium	Small	*F*	*P*
Bark pH	3.97±0.03a	4.00±0.03a	4.12±0.05b	**5.65**	**<0.001**
Bark roughness*	2.05±0.13a	1.85±0.13a	1.65±0.15a	2.06	0.147
Diameter at breast height (cm)	13.8±4.0a	12.4±4.0a	11.6±4.1a	2.70	0.086

Values given are Mean ± SE.

DF = 2, 27.

* Three level scale from smooth to very rough.

Letters distinguish significant differences (*P*<0.05, Tukey’s *post hoc* test).

### Study System

All islands consist of moraine deposits created when the land ice retreated 9000 years ago [30]. They vary in size from 0.02 to 15 ha and are grouped into three size classes; 10 ‘large’ (>1.0 ha), 10 ‘medium’ (0.1 to 1.0 ha) and 10 ‘small’ (<0.1 ha) [12], [31]. As such, the mean time since fire for the large, medium and small islands is 585, 2180 and 3250 years respectively. The vegetation of the large islands is dominated by early successional species (*Pinus sylvestris* and *Vaccinium myrtillus*), while small islands are dominated by late successional species (*Picea abies* and *Empetrum hermaphroditum*) [30]. *Betula pubescens* occurs commonly across the gradient, although as succession progresses the plants become smaller and less productive, and their foliar tissues become more defended [10], [12]. The atmospheric N deposition to this system is 2 kg ha^−1^ year^−1^ [37]. Further details about the environmental properties and traits of *B. pubescens* for each of the three island size classes are given in Table S1.

**Figure 2:**
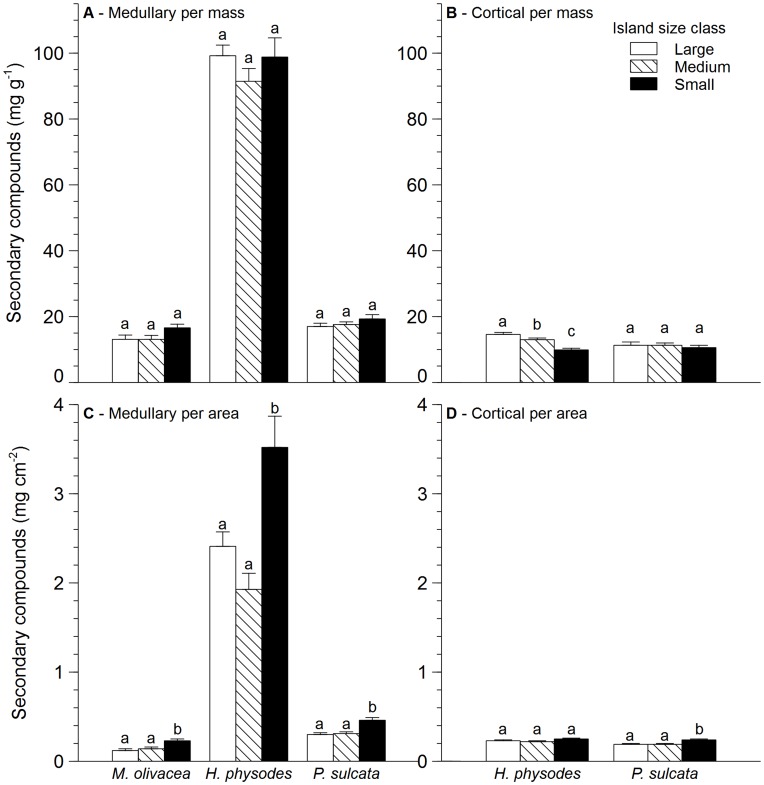
Response of lichen secondary compounds to island size. Values are given for concentrations (mean+SE) of medullary (A and C) and cortical (B and D) carbon based secondary compounds expressed per thallus mass (A–B) as well as per thallus area (C–D) in three lichen species across three island size classes. *Melanohalea olivacea* lacks cortical compounds and is therefore not included in panels B and D. Letters within species distinguish significant differences (*P*<0.05, Tukey’s *post hoc* test) between island size class.

### Lichen Material Collection and Analysis

One mature thallus of each of the three lichen species *H. physodes*, *M. olivacea* and *P. sulcata,* each measuring 2.1±0.75 cm^2^ (mean±SD), were collected at breast height on the northern side of each of four *Betula pubescens* trunks on each island; selected trees were between 8 and 20 cm diameter at breast height (DBH). We used only living trees close to plots with available environmental data collected from previous studies (Table S1) [12], [30], [31]. All plots were placed at similar distance from the shore to prevent edge and microclimatic effects from confounding the results [31]. Bark roughness and pH of the trunk surface was measured on epiphyte-free bark surfaces of each sampled tree in the vicinity of where the lichen material was collected. In line with other investigations [38], [39] that have used a three point scale to quantify bark roughness, we visually categorized bark roughness into 3 classes (smooth, some roughness and very rough surface). Bark pH was measured with a flat surface electrode pH meter (PH100: ExStick® pH meter) following spraying with 2 ml 0.1 M KCl solution as described by Farmer et al. [40].

Thalli were weighed air-dry (±0.1 mg) and thallus area following desiccation was measured with a leaf area meter (LI3100; LI-COR, Lincoln, Nebr., USA). Subsequently, the lichens were ground to powder with a ball mill and carbon based secondary compounds were extracted in 2 ml acetone for four 20 minute intervals, and the combined supernatants were evaporated to dryness and dissolved in 0.25–3 ml acetone. The extracted compounds were quantified by HPLC using an ODS Hypersil column, 50×4.6 mm using 0.25% orthophosphoric acid and 1.5% tetrahydrofuran in Millipore (Millipore, Billerica, Massachusetts, USA) water (A) and 100% methanol (B) as mobile phases at 2 ml/min, and UV detection at 245 nm, following Feige et al. [41] and Nybakken et al. [26]. Compound identification was based on retention times, online UV spectra and chromatography of standards. Each identified secondary compound was classified as either a medullary or a cortical compound based on the information presented in Thell and Moberg [42]. The total medullary and total cortical compounds in the thallus were then calculated both per unit thallus mass and per unit thallus area.

### Statistical Analyses

For each response variable for each island the mean value of the four thalli was determined and used as a single data value, so that individual islands served as the unit of replication. For the STM and total concentrations of medullary and cortical compounds, data was then analysed using split plot ANOVA with island size as the main plot factor and lichen species as the subplot factor. Further, the effect of island size on each CBSC for each lichen species, and on bark roughness and pH, was analysed by one way ANOVA. For each lichen species, Spearman rank correlation tests were used to determine the relationships of STM and concentrations of medullary and cortical compounds with environmental factors, with environmental factors consisting of bark roughness and pH, and measures of humus depth, light transmission, productivity and soil fertility determined for these islands from previous studies [5], [30], [31]. All analyses were performed in R 2.13.2.

## Results

Specific thallus mass of the three lichen species were approximately 45–60% higher on small islands than on large islands (Fig. 1, Table 1). There was a significant island size×species interaction due to lower, but still strong, effect of island size on STM for *M. olivacea* compared to the other two species. Specific thallus mass was positively correlated with bark pH, humus depth, soil N to P ratio and total N for all three species, and light for two of the three species. Meanwhile STM was negatively correlated with productivity for all species, and DBH and soil P for two of the species (Table 2). For the tree variables that we measured, bark pH was higher on small islands but there were no significant difference of DBH or bark roughness across the three island size classes (Table 3).

Medullary CBSCs per unit area were highest on small islands for all species (Fig. 2, Table 1). There was also a significant island size×species interaction, with the effect of island size being more pronounced for *H. physodes*. Meanwhile, medullary CBSCs per unit mass were not responsive to island size for any of the species. Cortical CBSCs only occurred in *H. physodes* and *P. sulcata*. Cortical CBSCs per unit mass were overall affected by island size, and there was a significant island size×species interaction because CBSCs for *H. physodes* declined with decreasing island size while those for *P. sulcata* were unresponsive (Fig. 2, Table 1). Meanwhile, for cortical CBSCs per unit area there was a significant effect of island size but no significant island size×species interaction; however only *P. sulcata* increased significantly with decreasing island size. Measures of STM were positively correlated with medullary CBSCs per unit area for all species, and with cortical CBSCs per unit area for *H. physodes* (Table 2). In contrast, STM was negatively correlated with cortical CBSCs per unit mass for *H. physodes* and *P. sulcata* (Table 2) and positively correlated with medullary CBSCs per unit mass for *M. olivacea* (0.550, P = 0.002). Total cortical CBSCs per unit area showed relatively few relationships with environmental variables, but for *H. physodes* cortical CBSCs per unit mass showed strong positive relationships with bark roughness, DBH, productivity and total soil P, and strong negative relationships with bark pH, humus depth, light and soil total N and N to P (Table 2). Medullary CBSCs per unit area showed several significant relationships; for all three species they were positively correlated with humus depth and soil N to P, and for two of the three species positively with bark pH and negatively with productivity and bark roughness (Table 2). Medullary CBSCs per unit mass were never significantly correlated with any environmental variables (data not presented).

When individual CBSCs were considered separately, both *H. physodes* and *P. sulcata* contained atranorin and chloroatranorin in the cortex (Table S2). *Hypogymnia physodes* contained three CBSCs in the medulla, i.e., protocetraric acid, physodic acid and physodalic acid. Meanwhile the other two species each only contained one medullary CBSC, i.e., salazinic acid for *P. sulcata* and fumarprotocetraric acid for *M. olivacea* (Table S2). For *H. physodes* the concentration of the physodic acid was lowest on medium size islands and concentrations of atranorin and chloroatranorin, increased with island size (Table S2). No other compounds were responsive to island size.

## Discussion

Specific thallus mass varied greatly across the island chronosequence. The small retrogressed islands, which have lower soil fertility and productivity [12], [31], and support smaller *B. pubescens* trees that are more defended [10] and that have a higher specific leaf mass (SLM) [11], also host lichens with higher specific thallus masses (STMs) and higher contents of CBSCs per unit area.

Specific thallus mass is often enhanced by increased light [15]–[18]. However, in our study the relationship of STM with light varied across species, being strongest for *P. sulcata* and non-significant for *M. olivacea.* Other factors, notably belowground properties indicative of soil fertility such as the soil N to P ratio, instead emerged as amongst the strongest predictors of lichen STM for all three species. This is consistent with previous work showing the soil N to P ratio to serve as a strong predictor of the SLM of vascular plants, both on these islands [11] and on other retrogressive chronosequences [8], [21]. This highlights the role of increasing soil N to P ratios during retrogression as an important driver of species traits of not just vascular plants, but also of other photosynthetic life forms such as lichens, and presumably ultimately the ecological processes regulated by these traits [6].

One interpretation for the observed increase in STM accompanying the increase in soil N to P ratio is that P limitation is restricting areal expansion of the lichen [43]. Meanwhile biomass growth per unit area is primarily related to net C gain [44], which is controlled by photobionts that are often more N-limited [45], and is strongly dependent on the amount of irradiation received by the wet thallus [44], [46]. Consequently, the increasing P limitation during retrogression would lead to a reduction in areal growth while biomass gain per unit area contributes to a higher STM. We also found that *M. olivacea* had the lowest STM of the three species for all island size classes, and also had the least plastic response to island size. This means that *M. olivacea* should have a low water holding capacity irrespective of environmental conditions [14], and consistent with this, desiccation has been shown to be an important regulator of the growth of this species [47]. We also found STM to be negatively but weakly related to soil pH (which frequently accompanies declining soil fertility) for *H. physodes* but not for the other species. This finding is consistent with [28] who found lower soil pH to contribute to increased STM for the cephalolichen *L. pulmonaria*. In contrast, we found a positive correlation of STM with bark pH for all three species, which was in line with our finding that bark pH increased as retrogression proceeded. This increase in bark pH is in contrast to the decline in soil pH and soil fertility that occurs as retrogression proceeds [30], and could be a result of the lichens, which are less abundant on small islands, lowering bark pH [48].

Medullary CBSCs per unit area increased during retrogression for all three species and cortical CBSCs per area increased for *P. sulcata*. However, retrogression did not increase CBSCs per unit mass, in contrast to what has been observed for secondary compounds in vascular plants [10], [29]. The observed increase in medullary CBSCs per area during ecosystem retrogression occurs because concentrations of CBSCs remain invariant across the gradient while thallus thickness and STM increases with decreasing island size. This also contributes to the strong relationship between STM and medullary CBSCs per unit area. Meanwhile, cortical CBSCs are situated in a thin layer within the lichen thallus. As such, cortical CBSCs per unit mass are more sensitive to dilution effects as the thickness of the medulla and therefore the whole thallus increases; this leads to declining concentrations of CBSCs with decreasing island size for *H. physodes* and negative relationships between CBSC concentrations and STM for both *H. physodes* and *P. sulcata*. Cortical CBSC are commonly expressed as per unit area rather than per unit mass, because this is more relevant to their role in conferring protection from adverse effects of light such as photoinhibition [24], [49]–[51]. In our study system, light transmission through the forest canopy is greatest on the small islands [31] and on these islands *P. sulcata* increases its production of atranorin and chloroatranorin per unit area, which should confer better protection against light. As such, atranorin has previously been shown to reduce photoinhibition [52].

We found support for our hypothesis that STM increases during retrogression, and this increase appears to be strongly linked to increasing soil N to P ratios for all three species. However, we found mixed support for our hypothesis that CBSCs will increase along this environmental gradient, because they increased during retrogression only when expressed per unit area. These findings contribute to our understanding of how the response of epiphyte traits to ecosystem retrogression covaries with that of their host plants. As such, they emphasize that retrogression causes predictable changes in traits of not just vascular plants [8]–[10] and mosses [13], but also of other photosynthesizing organisms such as lichens. Specific thallus mass and CBSCs could conceivably regulate lichen palatability and litter decomposition, in a manner similar to the role of SLM and phenolic compounds in vascular plants [19], [20], [53]. Both herbivory and decomposition are key processes driving the turnover of carbon and nutrients in terrestrial ecosystems [54]. Given the role that lichens can play in ecosystem nutrient cycling [35], [36], [55], variation in CBSCs and STM during retrogression could potentially have important implications for ecosystem-level processes that are yet to be explored.

## Supporting Information

Table S1 **Changes in selected ecosystem properties and traits of the host plant (** ***Betula pubescens*** **) across the island size gradient.**
(PDF)

Table S2 **Concentrentrations of individual carbon based secondary compounds in relation to island size.**
(PDF)
