# Supplementary material for: Lichen Specific Thallus Mass and Secondary Compounds Change across a Retrogressive Fire-Driven Chronosequence
Source: PLoS One. 2012 Nov 8;7(11):e49081. doi: 10.1371/journal.pone.0049081 (PMC3493489; doi:10.1371/journal.pone.0049081)
Supplement: Table S1 — Changes in selected ecosystem properties and traits of the host plant ( Betula pubescens ) across the island size gradient. (PDF) [file pone.0049081.s001.pdf]

Table S1. Changes in selected ecosystem properties and traits of the host plant (*Betula pubescens*) across the island size gradient. Values are means with standard errors in brackets Within each row numbers followed by the same letter are not statistically significant at $P = 0.05$ (Tukey's test following one-way ANOVA). Data from Wardle et al. (1997, 2003, 2004, 2012), Crutsinger et al. (2008) and Lagerström et al. (2012).

Ecosystem property	Island size		
	Small	Medium	Large
Humus depth (cm)	65.0 (4.7) a	41.4 (3.5) b	25.1 (3.5) c
Light transmission (%)	68.2 (2.6) a	46.7 (3.7) b	55.9 (4.5) a
Standing plant biomass (g/m^2/yr)	3470 (470) b	8340 (877) a	9349 (485) a
Net primary productivity (g/m^2/yr)	249 (24) b	368 (19) a	382 (23) a
Humus N (%)	1.42 (0.03) a	1.13 (0.04) ab	1.06 (0.04) b
Humus P (mg/g)	0.619 (0.024) b	0.684 (0.035) ab	0.754 (0.024) a
Humus N to P ratio	23.3 (1.1) a	19.1 (0.9) b	15.4 (0.5) c
Humus pH	3.38 (0.039) b	3.42 (0.027) ab	3.51 (0.029) a
Betula pubescens leaf traits:			
Specific leaf mass (mg/cm^2)	1.76 (0.06) a	1.54 (0.06) ab	1.51 (0.05) b
Leaf dry matter content (%)	43.2 (1.0) a	40.6 (0.8) ab	39.8 (0.7) b
Foliar N (%)	1.89 (0.06) b	2.09 (0.06) ab	2.11 (0.6) a
Cellulose (%)	14.1 (0.6) a	12.7 (0.4) ab	11.7 (0.8) b
Total phenolics (mg/g)	215.6 (4.9) a	199.0 (4.2) b	203.7 (2.5) ab
Gallic acid (mg/g)	2.20 (0.47) a	1.91 (0.52) ab	0.92 (0.14) b
Ellagic acid (mg/g)	23.21 (5.25) a	8.50 (1.79) b	2.97 (1.30) c
Net photosynthesis (nmol CO_2/g/s)	144 (5) b	170 (7) a	168 (9) a
