# Supplementary material for: Lichen Specific Thallus Mass and Secondary Compounds Change across a Retrogressive Fire-Driven Chronosequence
Source: PLoS One. 2012 Nov 8;7(11):e49081. doi: 10.1371/journal.pone.0049081 (PMC3493489; doi:10.1371/journal.pone.0049081)
Supplement: Table S2 — Concentrentrations of individual carbon based secondary compounds in relation to island size. (PDF) [file pone.0049081.s002.pdf]

**Table S2. Concentrentrations of individual carbon based secondary compounds in relation to island size**. Values given are mean ± SE mg $g^{-1}$ for each of three lichen species across three island size classes. Within each row numbers followed by the same letter are not statistically significant at *P* = 0.05 (Tukey's test following one-way ANOVA)

| Species / compound | Location in thallus | Large | Medium | Small | *F* | *P* |
|---|---|---|---|---|---|---|
| *Melanohalea olivacea* | | | | | | |
| Fumarprotocetraric acid | Medulla | 13.1±1.3 a | 13.1±1.2 a | 16.6±1.1 a | 2.74 | 0.083 |
| | | | | | | |
| *Hypogymnia physodes* | | | | | | |
| Protocetraric acid | Medulla | 63.9±2.7 a | 61.1±2.9 a | 64.0±4.4 a | 0.223 | 0.801 |
| Physodic acid | Medulla | 21.4±0.9 a | 17.5±0.9 b | 20.2±0.9 a | **4.55** | **0.020** |
| Physodalic acid | Medulla | 13.9±0.9 a | 12.8±0.6 a | 14.6±0.9 a | 1.22 | 0.310 |
| Atranorin | Cortex | 8.5±0.4 a | 7.4±0.3 b | 5.9±0.3 c | **14.05** | **<0.001** |
| Chloroatranorin | Cortex | 6.2±0.3 a | 5.6±0.2 a | 4.1±0.2 b | **20.12** | **<0.001** |
| | | | | | | |
| *Parmelia sulcata* | | | | | | |
| Salazinic acid | Medulla | 17.0±1.0 a | 17.6±0.8 a | 19.3±1.3 a | 1.30 a | 0.289 |
| Atranorin | Cortex | 5.4±0.5 a | 5.5±0.4 a | 5.7±0.3 a | 0.14 a | 0.869 |
| Chloroatranorin | Cortex | 5.9±0.5 a | 5.8±0.4 a | 4.9±0.4 a | 2.03 a | 0.151 |

DF = 2, 27
